# Ideal and actual partner assessments in male batterers with different attachment styles

**DOI:** 10.1371/journal.pone.0214388

**Published:** 2019-03-26

**Authors:** Rosaura Gonzalez-Mendez, Orlando Jiménez-Ardila, Gustavo Ramírez-Santana

**Affiliations:** 1 Departamento de Psicología Cognitiva, Social y Organizacional, Universidad de La Laguna, La Laguna, The Canary Islands, Spain; 2 Facultad de Psicología, Universidad Católica de Colombia, Bogotá, Colombia; 3 Departamento de Psicología Clínica, Psicobiología y Metodología, Universidad de La Laguna, La Laguna, The Canary Islands, Spain; Medical University of Vienna, AUSTRIA

## Abstract

Research analyzing male batterers’ views of what constitutes an ideal partner is scarce, and rejected features have not been tested. Analyzing the association of attraction and rejection patterns with attachment could help us understand how these men choose their female partners and the biases that make violence against these women more likely. The objective of this study was to analyze these patterns in male batterers with different attachment styles, considering both their ideal and actual partner assessments and the discrepancies between the two. Participants were 108 male offenders who were serving sentences in Colombian prisons for violence against women. In addition to identifying their attachment styles (secure, dismissive, preoccupied, and fearful), the study had participants assess their ideal and actual partners. The results showed significant differences in their actual partner assessments and in ideal-actual partner discrepancies, but not in their ideals. Secure attachment was related to the best partner assessments (higher scores in positive features and lower scores in negative ones), whereas the fearfully attached participants stood out for showing the worst assessments (higher scores in negative features and lower scores in positive ones). The findings provide evidence of the association of attachment styles with attraction and rejection patterns and offer suggestions for tailoring interventions.

## Introduction

Research on attraction has focused on analyzing those qualities that are preferred in a partner by most people, whereas rejected features have received little attention [1]. Preferences associated with gender and sexual orientation have also been examined [2, 3]. By contrast, relatively little research has studied the existence of discrepant attraction patterns in other groups. For instance, it has been found that teenagers with behavior problems [4] or people with rare attitudes [5] may differ from the majority in their preferences. However, very few studies have explored male batterers’ views of what constitutes their ideal partner [6] or the characteristics that they reject [1]. Examining these patterns may help us understand how these men choose the women who will become the targets of their abuse, as well as the biases that make intimate partner violence (IPV) more likely. Moreover, batterers are a heterogeneous group [7], and attachment dimensions have proven to be relevant to understanding the differences between them [8]. Hence, studying the association between attachment styles and attraction and rejection patterns may be useful to account for possible differences between batterers.

The interest in analyzing ideals about partners derives from their potential influence on the beginning and maintenance of intimate relationships. For instance, research has found that the match between ideals and a partner’s traits may predict relational outcomes when participants are actually in a relationship with the partner [9]. A large body of research on attraction has made it possible to identify the qualities that are the most attractive in a potential partner. Thus, positive characteristics such as intelligence, honesty, sense of humor or physical appeal are among the most desired traits across different ages and cultures [10, 11]. By contrast, only some studies have analyzed rejected features, even though they may have a greater influence on the evaluation of other people. Health problems, lack of hygiene or certain personality traits such as anger issues or being untrustworthy are among the worst-rated characteristics [1].

Although research focused specifically on intimate partner violent men’s preferences is scarce, those findings that are available point to the existence of patterns that differ from those of the general population. For example, Zayas and Shoda [12] found that male college students who reported psychological abuse toward their female partners showed greater preference than non-abusers for women who are anxiously attached and with low self-esteem and a history of victimization. Moreover, Jaspaert and Vervaeke [13] found that preference discrepancy (i.e., difference between partners’ ideal and real relationship) increased the likelihood of IPV perpetration. In a study with adolescents, the results showed that certain partner preferences and attachment styles moderate the link between witnessing IPV and dating violence [14]. Specifically, an increased risk of dating violence perpetration was detected among those boys who combined having witnessed IPV with a higher preference for rebellious and less good (e.g., less intelligent and honest) girls. Similarly, a preference for loving partners was found to predict dating violence perpetration among those highly avoidant boys who had witnessed IPV. Finally, findings also point to possible differences between batterers’ partner preferences, with those who are emotionally dependent interested in female characteristics that meet their psychological needs [6]. In this sense, attachment appears as a relevant factor to account for such differences.

Attachment theory is a useful framework for analyzing intimate relationships [15], and attachment styles have proven to be relevant in predicting IPV [8]. From this perspective, it is assumed that early negative experiences lead to the formation of internal working models that influence cognition, affect, and behavior in different relationships throughout life [16]. As expressions of those working models, attachment styles are usually measured through two dimensions: anxiety about abandonment and avoidance of intimacy [17, 18]. In addition, four attachment styles have been identified emerging from these two dimensions: secure (low in both dimensions), fearful (high in both dimensions), preoccupied (high in anxiety and low in avoidance), and dismissive (low in anxiety and high in avoidance) [19].

High levels of anxiety and/or avoidance are considered indicators of insecure attachment, which predicts numerous difficulties in intimate relationships. Specifically within abusive relationships, there is ample evidence linking insecure attachment to IPV, with batterers often showing high levels of anxiety and/or avoidance [20, 21]. In general, people with high avoidance tend to deactivate the attachment system, facilitating emotional distance in their relationships. Hence, batterers with this style of attachment are more likely to use violence to maintain emotional distance, but also as a way of exerting control or revenge [22]. By contrast, batterers who are highly anxious often feel unworthy of being loved and experience a high fear of abandonment. In this case, the relationship between insecure attachment and IPV seems to be better explained by separation anxiety and partner distrust [20].

### The current study

Violence against women is a serious and widespread problem worldwide [23], and Colombia is no exception. Nevertheless, the history of violence in this country has contributed to hindering the recognition of the problem and to delaying the search for solutions. Until 2012, men in Colombia accused of violence against their intimate partner were not necessarily prosecuted and imprisoned. With the agreement of the victim, the aggressor could opt for reconciliation, without reparation or compensation, after committing not to repeat the aggression and to improve his behavior within the family. Since 2013, conciliation has been prohibited in cases where women have filed an official complaint. Although these legislative changes have increased the protection of women, much remains to be done.

Reducing the recidivism of aggressors requires a better knowledge of the mechanisms that guide their behavior. Taking a step in this direction, the objective of this study was to analyze the attraction and rejection patterns in male batterers with different attachment styles. Specifically, we examined the preferred and rejected characteristics of their ideal partners, the actual partner assessments, and the discrepancies between the two measures. Given that batterers tend to show different attachment styles [8], we assumed they would show differences in their ideal and actual partner assessments. In this sense, our hypotheses were as follows:

Compared to the other batterers’ styles, we expected that the participants high in avoidant attachment (i.e., with dismissive and fearful styles) would show both a worse ideal partner (i.e., a lower preference for positive characteristics) and a higher rejection of undesirable characteristics, thus allowing greater emotional distance. More specifically, we expected they would show a lesser liking for Good (intelligent, honest, kind, and educated) and Affectionate (good wife, good mother, romantic, likes to practice sex, and good housekeeper) ideal partners and a greater preference for Rebellious ideal partners (likes to break the rules, rebellious, and with personality) (hypothesis 1). In addition, we expected a higher rejection of undesirable characteristics (unfaithful, liar, dirty, dishonest, etc.) (hypothesis 2).

By contrast, we expected that batterers who are low in avoidant attachment (secure and preoccupied) would make more positive evaluations of their actual partners than the other groups (hypothesis 3), i.e., giving higher scores in positive features (Good and Affectionate partners) and lower scores in negative ones (Rebellious and With Defects). In a similar vein, we also expected that secure and preoccupied participants would show a lesser discrepancy between their ideal and actual partner assessments than the other groups (hypothesis 4).

## Method

### Participants

Participants were 108 male offenders who were serving sentences in two Colombian prisons for violence against women. Their ages ranged from 20 to 60 (*M* = 35.3, *SD* = 10.2). Regarding their relationship status, 65.7% were married or cohabiting and 34.3% were single (51.3% said they were continuing their relationship while serving time in prison). The average length of their relationships was 8.6 years (*SD* = 7.6), and the majority had children (90.7%). In Colombia, socioeconomic level is officially identified through six strata that range from 1 (*the lowest*) to 6 (*the highest*). These strata are associated with the characteristics of people’s homes and the price that they have to pay for public services. According to this, 29.3% of the sample fell into stratum 1 (low-low), 40.5% into 2 (low), 24.2% into 3 (medium-low), and 6% into 4 (medium).

### Procedure

Compliance with ethical standards was positively assessed by both the Academic Committee of the Doctoral Program in Psychology and the Institutional Review Board of the corresponding author’s university (CEIBA). After receiving authorization from the Colombian prison authorities, we selected two prisons with a large number of men serving sentences for violence against their intimate partners. Once these specific prisoners had been identified, they were informed about the objective of the study and their voluntary participation was requested in writing. The anonymity and confidentiality of their responses were guaranteed in advance. The questionnaires did not include any information that would lead to the identification of the participants and the researchers retained these questionnaires at all times. Interviewers were trained to create an adequate rapport during data collection, which was carried out in suitable rooms.

### Measures

In addition to asking questions about demographic characteristics, we designed an instrument consisting of the different scales.

#### Attachment

The Spanish version of the Experiences in Close Relationships-Revised Scale (ECR-R) [17, 24] was used to measure two attachment dimensions: anxiety about abandonment and avoidance of intimacy. This short version of the scale consists of 18 items whose response options ranged from 1 (*strongly disagree*) to 7 (*strongly agree*). Internal consistency was calculated using Cronbach’s alpha, reaching values of .82 and .76, respectively.

#### Partner ideals

Preference for partner characteristics was measured through 12 items [14], which cover both desired traits and expected role. The instrument consists of three factors that depict, respectively, an Affectionate wife (good wife, good mother, likes to practice sex, romantic, and good housekeeper), a Good partner (intelligent, honest, kind, and educated), and a Rebellious partner (likes to break the rules, rebellious, and with personality). Response options ranged from 0 (*not at all*) to 10 (*very much*). Cronbach’s alphas were .81, .70, and .62, respectively.

#### Undesirable partner characteristics

To develop a measure of rejection of a potential female partner, we consulted different professionals who work with male batterers or female victims of IPV (*n* = 23). Based on their most frequent answers, 14 items were selected to assess the degree of male rejection of different negative characteristics (unfaithful, liar, dishonest, dirty, cold, emotionally unstable, rude, bad mother, defiant, not very intelligent, controlling, chatty, ugly, and submissive). As in the previous case, response options ranged from 0 (*not at all*) to 10 (*very much*). After testing that the *KMO* and the goodness of fit were adequate, exploratory factor analyses showed a single factor that accounted for 72% of variance (α = .97).

#### Actual partner assessment

Participants evaluated their actual partners according to the same characteristics used to rate their ideal partners (12 positive and 14 negative features). Response options ranged from 0 (*not at all*) to 10 (*very much*). Cronbach’s alphas obtained for the scales used to assess the positive characteristics reached values of .81 for the Good partner, .83 for the Affectionate wife, and .70 for the Rebellious partner. In the case of the Undesirable features, Cronbach’s alpha reached a value of .88.

### Data analysis

First, 50th percentile scores on anxiety and avoidance dimensions were determined separately, and participants were classified as either “low” (those who scored below the 50th percentile) or “high” (those who scored above the 50th percentile) in each of these two measures. Then, participants were classified into four groups according to their scores on each dimension: secure (*n* = 34), dismissive (*n* = 22), preoccupied (*n* = 26), and fearful (*n* = 26).

An ANOVA for each attraction (Good, Affectionate, and Rebellious) and rejection (With Defects) pattern was carried out to test the hypotheses. These patterns were first considered to test hypotheses 1 and 2, which refer to the ideal partner. Then, an ANOVA for each pattern was computed to test hypothesis 3, referring to the actual partner. Finally, an ANOVA was carried out to test hypothesis 4 (ideal-actual partner discrepancies). Orthogonal contrasts were performed as post-hoc comparisons in those evaluations that showed significant differences, to detect the differences between the attachment styles. The use of orthogonal contrasts as a post-hoc ANOVA test offers greater flexibility than the tests of mean differences. It allows for comparisons between individual means or between groups of means. In addition, it allows for total control in the estimation of errors α and β [25].

## Results

### Ideal and actual partner assessments

First, the analyses carried out with the preferred and rejected characteristics in an ideal partner did not show significant differences between batterers (Table 1). Therefore, these results failed to support hypotheses 1 and 2.

**Table 1:** ANOVAs for contrasting the ideal partner assessments in batterers with different attachment styles.

	Secure M (SD)	Dismissive M (SD)	Preoccupied M (SD)	Fearful M (SD)	F (3,107)	μp2
Affectionate	0.1 (0.5)	-0.2 (0.8)	-0.1 (1.6)	-0.0 (0.7)	0.5	0.01
Good	0.2 (0.9)	-0.4 (1.3)	0.2 (1.0)	-0.2 (1.0)	1.5	0.02
Rebellious	0.1 (1.1)	0.2 (0.8)	-0.2 (0.8)	-0.0 (1.1)	0.7	0.01
With Defects	0.0 (1.1)	-0.1 (0.9)	-0.1 (1.0)	0.0 (1.0)	0.2	0.00

Second, the results did show the expected differences when it came to assessing the actual partners, thus confirming hypothesis 3 (Table 2).

**Table 2:** ANOVAs for contrasting the actual partner assessments in batterers with different attachment styles.

	Secure M (SD)	Dismissive M (SD)	Preoccupied M (SD)	Fearful M (SD)	F (3,107)	μp2
Affectionate	0.5 (0.7)	-0.5 (1.1)	0.1 (0.8)	-0.3 (1.0)	6.8***	0.06
Good	0.4 (0.7)	-0.5 (1.2)	0.2 (0.7)	-0.2 (0.9)	4.9**	0.06
Rebellious	-0.4 (1.1)	0.1 (1.0)	0.3 (0.9)	0.3 (0.7)	3.9**	0.10
With Defects	-0.4 (1.1)	0.1 (1.0)	0.2 (1.0)	0.4 (0.8)	3.6**	0.09

**, *p* ≤ 0.010

***, *p* ≤ 0.001.

Consistently with their attachment styles, the secure participants showed the best scores in all the assessments, followed by the preoccupied participants. Specifically, the secure participants perceived their actual partners to be more affectionate and good than the dismissive and fearful participant groups did, and less rebellious and with fewer undesirable traits than the preoccupied and fearful groups did. In addition, the dismissive group rated their actual partners better than the preoccupied group did and as more affectionate than the fearful group (Table 3).

**Table 3:** Post-hoc analyses comparing the actual partner assessments in different pairs of attachment styles.

	Secure vs Dismissive	Secure vs Preoccupied	Secure vs Fearful	Dismissive vs Preoccupied	Dismissive vs Fearful	Preoccupied vs Fearful
	F (1,107) (μp2)	F (1,107) (μp2)	F (1,107) (μp2)	F (1,107) (μp2)	F (1,107) (μp2)	F (1,107) (μp2)
Affectionate	9.0** (0.08)	1.1 (0.01)	4.9* (0.05)	0.7 (0.01)	3.5* (0.04)	1.2 (0.01)
Good	16.8*** (0.14)	3.8 (0.04)	16.1*** (0.14)	4.4* (0.04)	0.1 (0.00)	3.7 (0.03)
Rebellious	3.4 (0.03)	7.4** (0.07)	10.4** (0.09)	0.5 (0.01)	1.3 (0.25)	0.2 (0.00)
With Defects	3.6 (0.03)	6.0* (0.05)	15.8*** (0.13)	0.2 (0.00)	3.2 (0.03)	2.0 (0.02)

*, *p* ≤ 0.050

**, *p* ≤ 0.010

***, *p* ≤ 0.001.

### Ideal-actual partner discrepancies

To test hypothesis 4, we calculated the participants’ ideal-actual partner discrepancies by subtracting the ideal and actual scores for each pattern (Affectionate, Good, Rebellious, and With Defects) separately (Table 4).

**Table 4:** ANOVAs for contrasting ideal-actual partner discrepancies in batterers with different attachment styles.

	Secure M (SD)	Dismissive M (SD)	Preoccupied M (SD)	Fearful M (SD)	F (3,107)	μp2
Affectionate	0.4 (0.6)	-0.4 (1.1)	0.1 (1.2)	-0.3 (1.0)	3.3*	0.06
Good	0.3 (0.9)	-0.3 (1.2)	0.8 (0.9)	-0.2 (0.7)	2.0	0.03
Rebellious	-0.5 (0.8)	-0.1 (1.2)	0.4 (0.9)	0.3 (1.1)	4.3**	0.09
With Defects	0.2 (1.0)	-0.1 (1.0)	-0.2 (0.9)	-0.1 (1.1)	1.0	0.03

*, *p* ≤ 0.050

**, *p* ≤ 0.010.

Significant differences were detected in the discrepancies of two patterns (Affectionate and Rebellious), partially supporting hypothesis 4. The secure participants showed a smaller discrepancy than the dismissive and fearful groups did when assessing the affectionate pattern, and a smaller discrepancy than the preoccupied and fearful groups when assessing rebelliousness (Table 5).

**Table 5:** Post-hoc analyses comparing the ideal-actual partner discrepancies in different pairs of attachment styles.

	Secure vs Dismissive	Secure vs Preoccupied	Secure vs Fearful	Dismissive vs Preoccupied	Dismissive vs Fearful	Preoccupied vs Fearful
	F (1,107) (μp2)	F (1,107) (μp2)	F (1,107) (μp2)	F (1,107) (μp2)	F (1,107) (μp2)	F (1,107) (μp2)
Affectionate	7.6** (0.07)	1.0 (0.01)	5.6* (0.06)	2.9 (0.03)	0.2 (0.00)	1.7 (0.02)
Rebellious	1.9 (0.02)	10.3** (0.09)	7.9** (0.08)	2.5 (0.02)	1.5 (0.02)	0.1 (0.00)

*, *p* ≤ 0.050

**, *p* ≤ 0.010.

## Discussion

The objective of this study was to analyze the attraction and rejection patterns in male batterers with different attachment styles. For this purpose, we considered their ideal and actual partner assessments, as well as the discrepancy between the two measures for each of the patterns analyzed (Good, Affectionate, Rebellious, and With Defects). As shown below, the results provide evidence that different attachment styles are associated with differences in both the actual partner assessments and the discrepancies between ideal and actual partner assessments, but not in the ideals.

Regarding ideals, the results failed to support an association with attachment styles (hypotheses 1 and 2). Specifically, participants did not show differences either in their preferred characteristics or in their rejected ones, depicting a homogeneous ideal of women. Although some studies have indicated that violent men differ from non-violent men in their partner ideals [12], differences in male batterers’ partner preferences and rejections had not been previously tested. In fact, Saunders et al. [6] found that emotionally dependent perpetrators seemed interested in female characteristics that fit their psychological needs, but it was not clear if those features are similar or not between batterers. In the current study, the participants did differ in their psychological needs, as indicated by their attachment styles, but they did not show differences when describing their ideals. However, batterers’ ideals could still be different from those shown by the general male population, as suggested by the evidence obtained with college students [12]. Since abusive and non-abusive young men differ in their preference for characteristics that make women more vulnerable (e.g., emotional dependence), it seems reasonable to expect that batterers also differ with respect to non-violent men. In this sense, being able to predict how violent men choose their partners would allow practitioners to warn women against starting abusive intimate relationships, which is especially relevant to prevent re-victimization.

By contrast, the results did show the expected differences when it came to evaluating the actual partners, thus confirming hypothesis 3. The secure participants stand out for their more positive partner assessments, followed by the preoccupied ones. According to the attachment framework, these results are consistent with lower avoidance, as well as the positive view of “the other” predicted by the attachment framework [19]. In the case of the securely attached participants, their partner assessments also seem to support a lower risk of recidivism, as found in non-pathological or family-only perpetrators [26].

Moreover, specific differences detected between the groups suggest emotional conflicts that are also consistent with attachment styles. For example, the assignment of rebelliousness to the actual partners distinguishes the secure batterers from those who are highly anxious (i.e., preoccupied and fearful), but not from the dismissive ones. For anxious batterers, attentional bias towards rejection signals may explain negative opinions, as well as explaining rumination and hostility [27].

The dismissive style is associated with less positive assessments (lower scores on affectionate and good patterns), but not with more negative ones (rebelliousness and undesirable characteristics). This is consistent with the emotional distance that dismissive individuals are thought to cultivate. By contrast, the fearful batterers stand out by attributing more undesirable traits than the others to their intimate partners, which is likely derived from their ambivalence.

The differences between batterers with respect to the ideal-actual partner discrepancies are also consistent with the attachment framework, partially supporting hypothesis 4. The discrepancy in the affectionate pattern discriminated between the secure participants and those high in avoidance (dismissive and fearful). This connects emotional distance, which is thought to characterize these two latter styles [20], with the perception that the intimate partners are less affectionate than expected. Moreover, the discrepancy in the rebelliousness pattern differentiated between the securely attached and the highly anxious (preoccupied and fearful) participants, who perceived less docility than expected in their intimate partners. Consistent with these results, Buck et al. [20] found that the association between insecure attachment and IPV can be mainly explained by separation anxiety and partner distrust.

As far as we know, this is the first study to analyze the attraction and rejection patterns in batterers with distinct attachment styles, confirming the existence of differences in partner assessments and in the discrepancies between ideal and actual partners. Although it is known that perpetrators tend to blame their victims, the findings of the current study contribute to better understanding the biases that mark their view of women. The attribution of negative features that elicit rejection tends to be greater among those with high avoidance, thus showing a greater emotional distance. Rebelliousness is especially attributed to women by those male batterers who are higher in anxious attachment, which may be accounted for by their ambivalent feelings. Overall, the secure batterers (low in anxiety and avoidance) stood out for showing the most positive and least discrepant evaluations, whereas the fearful batterers (high in both dimensions) showed the worst.

### Limitations and new directions of research

The current study has some limitations that make it impossible to rule out that batterers differ in their ideals, just as they do in their actual partner assessments. Thus, it is possible that the sample size was insufficient to find the hypothesized differences in ideals. Hence, there is a need to analyze this association in larger samples. In addition, it would be necessary to examine these patterns in batterers who are receiving intervention in the community, since lack of direct contact with women in prison may have contributed to homogenizing their ideal partner assessments in a stereotyped way.

Although the attachment styles have proven useful for capturing the variability of IPV perpetrators, the link between insecure attachment and battering may be mediated by other factors, such as dysfunctional personality traits [8]. Therefore, a more complete analysis of possible differences in batterers’ ideals requires examining their personality profiles, which continue to form the basis of different batterer typologies [28, 29].

The cross-sectional design of this study does not allow us to confirm if the batterers with the best partner assessments (higher scores in positive features and lower scores in negative ones) are really those who show a lower recidivism. In this sense, a longitudinal design would be necessary to determine if there is less recidivism in this group of batterers.

### Implications for intervention

Meta-analyses of the effectiveness of batterer intervention programs have revealed a limited effect [30, 31, 32]. However, there is agreement that effectiveness depends on the extent to which treatments are tailored to the batterer profile [33, 34]. High levels of insecure attachment observed in male offenders suggest the usefulness of incorporating the attachment perspective when designing therapeutic objectives and professionals may consider some clues for clinical purposes [35, 36]. In addition, although the scale used in this study has proven to be effective in predicting the batterers’ assessments with different attachment styles, practitioners may assess the usefulness of other measures to expand the information on the batterers’ attachment representations [37, 38, 39].

Secure base priming has proven effective in reducing experimentally produced anger [40]. Interventions based on the attachment perspective try to reinforce the sense of security within a therapeutic relationship [36]. However, prison often become a re-enactment of the offenders’ early attachment experiences, which makes intervention in the community more effective [41]. In addition to reinforcing the need to address insecure attachment in batterer intervention programs, the findings of the current study may help adapt these interventions by establishing more complete offender profiles. Emotional problems, denial, minimization, and blame are among the challenges to promoting behavioral change that have been identified by practitioners [42]. The differences found between the participants with distinct attachment styles may guide practitioners to make perpetrators aware of their ineffective coping strategies, as well as the origin and consequences of their partner views. In a similar vein, it has been suggested that there is a need to improve their mentalization capacity (i.e. the ability to understand mental states in themselves and others) as a way to increase affect regulation [41].

This is one of the few studies carried out with batterers in Colombia, a little-known cultural context for researchers interested in this research area. Despite the high levels of violence that have characterized this country, the findings are consistent with the previous literature that indicates the importance of considering attachment styles in batterer intervention programs. In this sense, education about the cognitions, affect, and behaviors associated with the internal working models of the different attachment styles needs to be considered. Moreover, batterer intervention programs are not well developed in Colombia, and the findings of this study may promote their implementation in the community among those who do not have other criminal records.

In short, the findings provide evidence of the association of attachment styles with attraction and rejection patterns, which may be useful to design differentiated treatment approaches tailored to the offender. The batterers’ classification into four attachment styles allowed us to find significant differences in their actual partner assessments and in ideal-actual partner discrepancies that are consistent with what was expected.

## Supporting information

S1 Questionnaire Spanish version of the questionnaire is the S1 Questionnaire legend. (DOCX)

S2 Questionnaire English version of the questionnaire is the S2 Questionnaire legend. (DOCX)

S1 File DataBase is the S1 File legend. (XLSX)
