# Supplementary material for: Ideal and actual partner assessments in male batterers with different attachment styles
Source: PLoS One. 2019 Mar 26;14(3):e0214388. doi: 10.1371/journal.pone.0214388 (PMC6435157; doi:10.1371/journal.pone.0214388)
Supplement: S1 Questionnaire — (DOCX) [file pone.0214388.s001.docx]

Spanish version of the Questionnaire

| Edad |  | Estado civil |  | Estatus socioeconómico | |  |
| --- | --- | --- | --- | --- | --- | --- |
| Estatus de su relación de pareja |  | Tiempo de relación |  | Número de hijos | |  |
|  |  |  |  | Fecha de Aplicación |  | |

| ¿Cómo es su pareja ideal?  Por favor, responda de 0 (*nada)* a 10 (*mucho*) | 0 | 1 | 2 | 3 | 4 | 5 | 6 | 7 | 8 | 9 | 10 |
| --- | --- | --- | --- | --- | --- | --- | --- | --- | --- | --- | --- |
| Buena esposa |  |  |  |  |  |  |  |  |  |  |  |
| Buena madre |  |  |  |  |  |  |  |  |  |  |  |
| Le gusta las relaciones sexuales |  |  |  |  |  |  |  |  |  |  |  |
| Romántica |  |  |  |  |  |  |  |  |  |  |  |
| Buena ama de casa |  |  |  |  |  |  |  |  |  |  |  |
| Inteligente |  |  |  |  |  |  |  |  |  |  |  |
| Honesta |  |  |  |  |  |  |  |  |  |  |  |
| Amable |  |  |  |  |  |  |  |  |  |  |  |
| Educada |  |  |  |  |  |  |  |  |  |  |  |
| Le gusta saltarse las normas |  |  |  |  |  |  |  |  |  |  |  |
| Rebelde |  |  |  |  |  |  |  |  |  |  |  |
| Con personalidad |  |  |  |  |  |  |  |  |  |  |  |

| ¿Qué es lo que no  soporta en una pareja?  Por favor, responda de 0 (*nada)* a 10 (*mucho*) | 0 | | 1 | | 2 | | 3 | | 4 | | 5 | | 6 | | 7 | | 8 | | 9 | | 10 | |  |
| --- | --- | --- | --- | --- | --- | --- | --- | --- | --- | --- | --- | --- | --- | --- | --- | --- | --- | --- | --- | --- | --- | --- | --- |
| Infiel |  | |  | |  | |  | |  | |  | |  | |  | |  | |  | |  | |  |
| Mentirosa |  | |  | |  | |  | |  | |  | |  | |  | |  | |  | |  | |  |
| Deshonesta |  | |  | |  | |  | |  | |  | |  | |  | |  | |  | |  | |  |
| Sucia |  | |  | |  | |  | |  | |  | |  | |  | |  | |  | |  | |  |
| Fría |  | |  | |  | |  | |  | |  | |  | |  | |  | |  | |  | |  |
| Emocionalmente inestable |  | |  | |  | |  | |  | |  | |  | |  | |  | |  | |  | |  |
| Grosera |  | |  | |  | |  | |  | |  | |  | |  | |  | |  | |  | |  |
| Mala madre |  | |  | |  | |  | |  | |  | |  | |  | |  | |  | |  | |  |
| Desafiante |  | |  | |  | |  | |  | |  | |  | |  | |  | |  | |  | |  |
| Poco inteligente |  | |  | |  | |  | |  | |  | |  | |  | |  | |  | |  | |  |
| Controladora |  | |  | |  | |  | |  | |  | |  | |  | |  | |  | |  | |  |
| Que hable mucho |  | |  | |  | |  | |  | |  | |  | |  | |  | |  | |  | |  |
| Fea |  | |  | |  | |  | |  | |  | |  | |  | |  | |  | |  | |  |
| Sumisa |  | |  | |  | |  | |  | |  | |  | |  | |  | |  | |  | |  |
| ¿Cómo es su pareja real?  Por favor, responda de 0 (*nada)* a 10 (*mucho*) | | 0 | | 1 | | 2 | | 3 | | 4 | | 5 | | 6 | | 7 | | 8 | | 9 | | 10 | |
| Buena esposa | |  | |  | |  | |  | |  | |  | |  | |  | |  | |  | |  | |
| Buena madre | |  | |  | |  | |  | |  | |  | |  | |  | |  | |  | |  | |
| Le gusta las relaciones sexuales | |  | |  | |  | |  | |  | |  | |  | |  | |  | |  | |  | |
| Romántica | |  | |  | |  | |  | |  | |  | |  | |  | |  | |  | |  | |
| Buena ama de casa | |  | |  | |  | |  | |  | |  | |  | |  | |  | |  | |  | |
| Inteligente | |  | |  | |  | |  | |  | |  | |  | |  | |  | |  | |  | |
| Honesta | |  | |  | |  | |  | |  | |  | |  | |  | |  | |  | |  | |
| Amable | |  | |  | |  | |  | |  | |  | |  | |  | |  | |  | |  | |
| Educada | |  | |  | |  | |  | |  | |  | |  | |  | |  | |  | |  | |
| Le gusta saltarse las normas | |  | |  | |  | |  | |  | |  | |  | |  | |  | |  | |  | |
| Rebelde | |  | |  | |  | |  | |  | |  | |  | |  | |  | |  | |  | |
| Con personalidad | |  | |  | |  | |  | |  | |  | |  | |  | |  | |  | |  | |
| Infiel | |  | |  | |  | |  | |  | |  | |  | |  | |  | |  | |  | |
| Mentirosa | |  | |  | |  | |  | |  | |  | |  | |  | |  | |  | |  | |
| Deshonesta | |  | |  | |  | |  | |  | |  | |  | |  | |  | |  | |  | |
| Sucia | |  | |  | |  | |  | |  | |  | |  | |  | |  | |  | |  | |
| Fría | |  | |  | |  | |  | |  | |  | |  | |  | |  | |  | |  | |
| Emocionalmente inestable | |  | |  | |  | |  | |  | |  | |  | |  | |  | |  | |  | |
| Grosera | |  | |  | |  | |  | |  | |  | |  | |  | |  | |  | |  | |
| Mala madre | |  | |  | |  | |  | |  | |  | |  | |  | |  | |  | |  | |
| Desafiante | |  | |  | |  | |  | |  | |  | |  | |  | |  | |  | |  | |
| Poco inteligente | |  | |  | |  | |  | |  | |  | |  | |  | |  | |  | |  | |
| Controladora | |  | |  | |  | |  | |  | |  | |  | |  | |  | |  | |  | |
| Que hable mucho | |  | |  | |  | |  | |  | |  | |  | |  | |  | |  | |  | |
| Fea | |  | |  | |  | |  | |  | |  | |  | |  | |  | |  | |  | |
| Sumisa | |  | |  | |  | |  | |  | |  | |  | |  | |  | |  | |  | |

| En general ¿cómo describiría  sus relaciones de pareja?  Por favor, responda de 1 (si está *total desacuerdo*) a 7 (si está *total acuerdo*) | 1 | 2 | 3 | 4 | 5 | 6 | 7 |
| --- | --- | --- | --- | --- | --- | --- | --- |
| Me siento incómodo cuando mi pareja quiere mucha cercanía. |  |  |  |  |  |  |  |
| Mis relaciones me generan mucha ansiedad y preocupación. |  |  |  |  |  |  |  |
| Hablo las cosas con mi pareja. |  |  |  |  |  |  |  |
| Cuando muestro mis sentimientos, tengo  miedo de que ella no sienta lo mismo por mí. |  |  |  |  |  |  |  |
| Me preocupa que mi pareja no se preocupe  tanto por mí como lo hago yo por ella. |  |  |  |  |  |  |  |
| Me siento cómodo compartiendo  mis pensamientos y sentimientos más personales con mi pareja. |  |  |  |  |  |  |  |
| En momentos de necesidad, me ayuda poder contar con mi pareja. |  |  |  |  |  |  |  |
| Me asusta no gustarle a una pareja cuando sepa cómo soy. |  |  |  |  |  |  |  |
| Me preocupa no estar a la altura. |  |  |  |  |  |  |  |
| Le cuento a mi pareja prácticamente todo. |  |  |  |  |  |  |  |
| Prefiero no tener demasiada cercanía e intimidad emocional con mi pareja. |  |  |  |  |  |  |  |
| Habitualmente comento mis problemas y preocupaciones a mi pareja. |  |  |  |  |  |  |  |
| Mi pareja realmente me comprende y conoce mis necesidades. |  |  |  |  |  |  |  |
| Encuentro relativamente fácil tener mucha cercanía e intimidad afectiva con mi pareja. |  |  |  |  |  |  |  |
| A menudo me produce ansiedad que mi pareja realmente no me ame. |  |  |  |  |  |  |  |
| A menudo desearía que los sentimientos de mi pareja  hacia mí fueran tan fuertes como mis sentimientos por ella. |  |  |  |  |  |  |  |
| Cuando mi pareja no está cerca, me preocupa que ella pueda llegar a estar interesada en alguien más |  |  |  |  |  |  |  |
| Con frecuencia me preocupa que mi pareja no quiera estar conmigo |  |  |  |  |  |  |  |
