# Supplementary material for: Ideal and actual partner assessments in male batterers with different attachment styles
Source: PLoS One. 2019 Mar 26;14(3):e0214388. doi: 10.1371/journal.pone.0214388 (PMC6435157; doi:10.1371/journal.pone.0214388)
Supplement: S2 Questionnaire — (DOCX) [file pone.0214388.s002.docx]

English version of the Questionnaire

| Age |  | Marital status |  | Socieconomic status | |  |
| --- | --- | --- | --- | --- | --- | --- |
| Status of your relationship |  | Relationship length |  | Number of children | |  |
|  |  |  |  | Date |  | |

| What is your ideal partner like?  Please answer from 0 (*not at all*) to 10 (*very much*) | 0 | 1 | 2 | 3 | 4 | 5 | 6 | 7 | 8 | 9 | 10 |
| --- | --- | --- | --- | --- | --- | --- | --- | --- | --- | --- | --- |
| Good wife |  |  |  |  |  |  |  |  |  |  |  |
| Good mother |  |  |  |  |  |  |  |  |  |  |  |
| Likes to practice sex |  |  |  |  |  |  |  |  |  |  |  |
| Romantic |  |  |  |  |  |  |  |  |  |  |  |
| Good housekeeper |  |  |  |  |  |  |  |  |  |  |  |
| Intelligent |  |  |  |  |  |  |  |  |  |  |  |
| Honest |  |  |  |  |  |  |  |  |  |  |  |
| Kind |  |  |  |  |  |  |  |  |  |  |  |
| Educated |  |  |  |  |  |  |  |  |  |  |  |
| Likes to break the rules |  |  |  |  |  |  |  |  |  |  |  |
| Rebellious |  |  |  |  |  |  |  |  |  |  |  |
| With personality |  |  |  |  |  |  |  |  |  |  |  |

| What can you not stand in a partner?  Please answer from 0 (*not at all*) to 10 (*very much*) | 0 | | 1 | | 2 | | 3 | | 4 | | 5 | | 6 | | | 7 | | | | 8 | | | 9 | | | 10 | | | |  |  |
| --- | --- | --- | --- | --- | --- | --- | --- | --- | --- | --- | --- | --- | --- | --- | --- | --- | --- | --- | --- | --- | --- | --- | --- | --- | --- | --- | --- | --- | --- | --- | --- |
| Unfaithful |  | |  | |  | |  | |  | |  | |  | | |  | | | |  | | |  | | |  | | | |  |  |
| Liar |  | |  | |  | |  | |  | |  | |  | | |  | | | |  | | |  | | |  | | | |  |  |
| Dishonest |  | |  | |  | |  | |  | |  | |  | | |  | | | |  | | |  | | |  | | | |  |  |
| Dirty |  | |  | |  | |  | |  | |  | |  | | |  | | | |  | | |  | | |  | | | |  |  |
| Cold |  | |  | |  | |  | |  | |  | |  | | |  | | | |  | | |  | | |  | | | |  |  |
| Emotionally unstable |  | |  | |  | |  | |  | |  | |  | | |  | | | |  | | |  | | |  | | | |  |  |
| Rude |  | |  | |  | |  | |  | |  | |  | | |  | | | |  | | |  | | |  | | | |  |  |
| Bad mother |  | |  | |  | |  | |  | |  | |  | | |  | | | |  | | |  | | |  | | | |  |  |
| Defiant |  | |  | |  | |  | |  | |  | |  | | |  | | | |  | | |  | | |  | | | |  |  |
| Not very intelligent |  | |  | |  | |  | |  | |  | |  | | |  | | | |  | | |  | | |  | | | |  |  |
| Controlling |  | |  | |  | |  | |  | |  | |  | | |  | | | |  | | |  | | |  | | | |  |  |
| Chatty |  | |  | |  | |  | |  | |  | |  | | |  | | | |  | | |  | | |  | | | |  |  |
| Ugly |  | |  | |  | |  | |  | |  | |  | | |  | | | |  | | |  | | |  | | | |  |  |
| Submissive |  | |  | |  | |  | |  | |  | |  | | |  | | | |  | | |  | | |  | | | |  |  |
| How would you describe  your actual partner?  Please answer from 0 (*not at all*) to 10 (*very much*) | | 0 | | 1 | | 2 | | 3 | | 4 | | 5 | | 6 | | | | 7 | | | 8 | | | 9 | | | 10 | | | |  |
| Good wife | |  | |  | |  | |  | |  | |  | |  | | | |  | | |  | | |  | | |  | | | |  |
| Good mother | |  | |  | |  | |  | |  | |  | |  | | | |  | | |  | | |  | | |  | | | |  |
| Likes to practice sex | |  | |  | |  | |  | |  | |  | |  | | | |  | | |  | | |  | | |  | | | |  |
| Romantic | |  | |  | |  | |  | |  | |  | |  | | | |  | | |  | | |  | | |  | | | |  |
| Good housekeeper | |  | |  | |  | |  | |  | |  | |  | | | |  | | |  | | |  | | |  | | | |  |
| Intelligent | |  | |  | |  | |  | |  | |  | |  | | | |  | | |  | | |  | | |  | | | |  |
| Honest | |  | |  | |  | |  | |  | |  | |  | | | |  | | |  | | |  | | |  | | | |  |
| Kind | |  | |  | |  | |  | |  | |  | |  | | | |  | | |  | | |  | | |  | | | |  |
| Educated | |  | |  | |  | |  | |  | |  | |  | | | |  | | |  | | |  | | |  | | | |  |
| Likes to break the rules | |  | |  | |  | |  | |  | |  | |  | | | |  | | |  | | |  | | |  | | | |  |
| Rebellious | |  | |  | |  | |  | |  | |  | |  | | | |  | | |  | | |  | | |  | | | |  |
| With personality | |  | |  | |  | |  | |  | |  | |  | | | |  | | |  | | |  | | |  | | | |  |
| Unfaithful | |  | |  | |  | |  | |  | |  | |  | | | |  | | |  | | |  | | |  | | | |  |
| Liar | |  | |  | |  | |  | |  | |  | |  | | | |  | | |  | | |  | | |  | | | |  |
| Dishonest | |  | |  | |  | |  | |  | |  | |  | | | |  | | |  | | |  | | |  | | | |  |
| Dirty | |  | |  | |  | |  | |  | |  | |  | | | |  | | |  | | |  | | |  | | | |  |
| Cold | |  | |  | |  | |  | |  | |  | |  | | | |  | | |  | | |  | | |  | | | |  |
| Emotionally unstable | |  | |  | |  | |  | |  | |  | |  | | | |  | | |  | | |  | | |  | | | |  |
| Rude | |  | |  | |  | |  | |  | |  | |  | | | |  | | |  | | |  | | |  | | | |  |
| Bad mother | |  | |  | |  | |  | |  | |  | |  | | | |  | | |  | | |  | | |  | | | |  |
| Defiant | |  | |  | |  | |  | |  | |  | |  | | | |  | | |  | | |  | | |  | | | |  |
| Not very intelligent | |  | |  | |  | |  | |  | |  | |  | | | |  | | |  | | |  | | |  | | | |  |
| Controlling | |  | |  | |  | |  | |  | |  | |  | | | |  | | |  | | |  | | |  | | | |  |
| Chatty | |  | |  | |  | |  | |  | |  | |  | | | |  | | |  | | |  | | |  | | | |  |
| Ugly | |  | |  | |  | |  | |  | |  | |  | | | |  | | |  | | |  | | |  | | | |  |
| Submissive | |  | |  | |  | |  | |  | |  | |  | | | |  | | |  | | |  | | |  | | | |  |
| In general, how would you describe your partner relationships?  Please answer from 1 (if you *strongly disagree*) to 7 (if you *strongly agree*) | | | | | | | | | | | | | | | 1 | | 2 | | 3 | | | 4 | | | 5 | | | 6 | 7 | | |
| I get uncomfortable when a romantic partner wants to be very close. | | | | | | | | | | | | | | |  | |  | |  | | |  | | |  | | |  |  | | |
| I worry a lot about my relationships. | | | | | | | | | | | | | | |  | |  | |  | | |  | | |  | | |  |  | | |
| I talk things over with my partner. | | | | | | | | | | | | | | |  | |  | |  | | |  | | |  | | |  |  | | |
| When I show my feelings for romantic partners, I'm afraid they will not feel the same about me. | | | | | | | | | | | | | | |  | |  | |  | | |  | | |  | | |  |  | | |
| I worry that my romantic partner won’t care about me as much as I care about her. | | | | | | | | | | | | | | |  | |  | |  | | |  | | |  | | |  |  | | |
| I feel comfortable sharing my private thoughts and feelings. | | | | | | | | | | | | | | |  | |  | |  | | |  | | |  | | |  |  | | |
| It helps to turn to my romantic partner in times of need. | | | | | | | | | | | | | | |  | |  | |  | | |  | | |  | | |  |  | | |
| I'm afraid that once a romantic partner gets to know me, she won't like who I really am. | | | | | | | | | | | | | | |  | |  | |  | | |  | | |  | | |  |  | | |
| I worry that I won't measure up to other people. | | | | | | | | | | | | | | |  | |  | |  | | |  | | |  | | |  |  | | |
| I tell my partner just about everything. | | | | | | | | | | | | | | |  | |  | |  | | |  | | |  | | |  |  | | |
| I prefer not to be too close to my romantic partner. | | | | | | | | | | | | | | |  | |  | |  | | |  | | |  | | |  |  | | |
| I usually discuss my problems and concerns with my partner. | | | | | | | | | | | | | | |  | |  | |  | | |  | | |  | | |  |  | | |
| My partner really understands my needs and me. | | | | | | | | | | | | | | |  | |  | |  | | |  | | |  | | |  |  | | |
| I find it relatively easy to get close to my partner. | | | | | | | | | | | | | | |  | |  | |  | | |  | | |  | | |  |  | | |
| I often worry that my partner does not really love me. | | | | | | | | | | | | | | |  | |  | |  | | |  | | |  | | |  |  | | |
| I often wish that my partner's feelings for me were as strong as my feelings for her. | | | | | | | | | | | | | | |  | |  | |  | | |  | | |  | | |  |  | | |
| When my partner is out of sight, I worry that he or she might become interested in someone else. | | | | | | | | | | | | | | |  | |  | |  | | |  | | |  | | |  |  | | |
| I often worry that my partner will not want to stay with me. | | | | | | | | | | | | | | |  | |  | |  | | |  | | |  | | |  |  | | |
